# Synthesis, crystal structure and Hirshfeld surface analysis of (4-methyl­phen­yl)[1-(penta­fluoro­phen­yl)-5-(tri­fluoro­meth­yl)-1*H*-1,2,3-triazol-4-yl]methanone

**DOI:** 10.1107/S2056989021010070

**Published:** 2021-10-05

**Authors:** Nazariy T. Pokhodylo, Yurii Slyvka, Evgeny Goreshnik, Roman Lytvyn

**Affiliations:** a Department of Organic Chemistry, Ivan Franko National University of Lviv, Kyryla i Mefodiya, 6, Lviv, 79005, Ukraine; b Department of Inorganic Chemistry, Ivan Franko National University of Lviv, Kyryla i Mefodiya, 6, Lviv, 79005, Ukraine; c Department of Inorganic Chemistry and Technology, Jozef Stefan Institute, Jamova 39, SI-1000 Ljubljana, Slovenia

**Keywords:** crystal structure, 1,2,3-triazole, Hirshfeld surface analysis

## Abstract

The title compound, C_17_H_7_F_8_N_3_O, was obtained *via* the reaction of 1-azido-2,3,4,5,6-penta­fluoro­benzene with 4,4,4-tri­fluoro-1-(*p*-tol­yl)butane-1,3-dione using tri­ethyl­amine as a base catalyst and solvent. The dihedral angles between the penta­fluoro­phenyl (*A*), triazole (*B*) and *p*-tolyl (*C*) rings are *A*/*B* = 62.3 (2), *B*/*C* = 43.9 (3) and *A*/*C* = 19.1 (3)°. In the crystal, the mol­ecules are linked by C—H⋯F and C—H⋯O hydrogen bonds as well as by aromatic π–π stacking inter­actions into a three-dimensional network. To further analyse the inter­molecular inter­actions, a Hirshfeld surface analysis was performed.

## Abstract 2

The title compound was obtained *via* the reaction of 1-azido-2,3,4,5,6-penta­fluoro­benzene with 4,4,4-tri­fluoro-1-(*p*-tol­yl)butane-1,3-dione using tri­ethyl­amine as a base catalyst and solvent. In the crystal, the mol­ecules are linked by C—H⋯F and C—H⋯O hydrogen bonds as well as by aromatic π–π stacking inter­actions into a three-dimensional network.

## Chemical context

Compounds with perfluoro­aromatic motifs are of inter­est for the design of fluorescence materials, including their application in optoelectronic devices (Funabiki *et al.*, 2021 ▸; Feng *et al.*, 2021 ▸; Moseev *et al.*, 2019 ▸; Kandhadi *et al.*, 2018 ▸; Lukeš *et al.*, 2016 ▸; Wang *et al.*, 2013 ▸; Matsui *et al.*, 2008 ▸). For instance, the perfluoro­biphenyl moiety was used as an electron acceptor for new donor–acceptor compounds with thermally activated delayed fluorescence (TADF) applied for the fabrication of TADF-based OLEDs (Danyliv *et al.*, 2021 ▸; Hladka *et al.*, 2018 ▸). On the other hand, 1,2,3-triazoles, as a result of their electron-accepting properties, are widely used in the design of organic phosphors (Gavlik *et al.*, 2017 ▸; Fernández-Hernández *et al.*, 2013 ▸; Tomkute-Luksiene *et al.*, 2013 ▸; Ichikawa *et al.*, 2011 ▸). Recently, a series of 3,6-bis­(4-triazol­yl)pyridazines equipped with terminal phenyl substituents with varying degrees of fluorination were synthesized and proposed to be used as electron-transporting/hole-blocking materials in organic electronics (Birkenfelder *et al.*, 2017 ▸). In view of this, we decided to combine these two fragments in order to construct new mol­ecular scaffolds of compounds that have the potential for use in optoelectronic devices.

Despite the prospects of using 1-(perfluoro­phen­yl)-1*H*-1,2,3-triazole in the creation of phosphor materials, the paths of their synthesis are poorly studied. It is known that azides are convenient precursors of 1,2,3-triazoles. A literature survey showed limited data on the reaction of perfluoro­phenyl­azide in the synthesis of 1,2,3-triazoles. The reactions of such azides with acetyl­enes, which occur as a 1,3-dipolar cyclo­addition, are primarily studied. For example, perfluoro­phenyl­azide was studied in the copper-catalysed azide–alkyne cyclo­addition (CuAAC) click reaction with propargyl alcohol (Lavoie *et al.*, 2017 ▸), 5-chloro­pent-1-yne and 6-chloro­hex-1-yne (Berry *et al.*, 2014 ▸), trimeth­yl[(perfluoro­phen­yl)ethyn­yl]silane (Lu *et al.*, 2012 ▸), iodo­ethynylarenes (Maugeri *et al.*, 2016 ▸), 4-ethynyl­phospholo[3,2-*b*:4,5-*b*′]di­thio­phene 4-oxide (He, Zhang *et al.*, 2013 ▸), [4-(iodo­ethyn­yl)phen­yl]di­phenyl­phosphine oxide (Maugeri *et al.*, 2017 ▸), 2-ethynyl­pyridine (Liu *et al.*, 2011 ▸), bis-alkynes (Milo *et al.*, 2015 ▸) and 2,8-diethynyl-5-phenyl-4*H*-phosphepino[4,3-*b*:5,6-*b*′]di­thio­phene-4,6(5*H*)-di­one (He, Borau-Garcia *et al.*, 2013 ▸). The CuAAC reaction of perfluoro­phenyl­azide was used for the synthesis and bioactivity of phthalimide analogues as potential drugs to treat schistosomiasis (Singh *et al.*, 2020 ▸) and for identification of sialoside analogues for siglec-based cell targeting (Rillahan *et al.*, 2012 ▸). Moreover, the 1,3-dipolar cyclo­addition of perfluorinated aryl azides with enamines and strained dipolar­ophiles has been studied (Xie *et al.*, 2015 ▸). Additionally, non-catalytic Huisgen (3 + 2) cyclo­addition of per­fluoro­phenyl­azide with ethyl propiolate and a one-pot tandem Sonogashira cross-coupling/CuAAC reaction were studied (Kloss *et al.*. 2011 ▸). Conversely, for the synthesis of fully substituted 1,2,3-triazoles, Dimroth-type reactions are the most convenient. However, there is only one example of base-promoted cyclization of perfluoro­phenyl­azide with methyl­ene active ketones (Dimroth-type reaction) in the triazole synthesis. Thus, by the reaction of perfluoro­phenyl­azide with acetyl­acetone in CHCl_3_ under Et_3_N and DBU catalysis, 1,2,3-triazoles were formed in 57% yield (Shafran *et al.*, 2019 ▸). It should be noted that the classical conditions of the Dimroth reaction are MeONa/MeOH (Krivopalov *et al.*, 2005 ▸). Such conditions are suitable for the rapid formation of polyheterocyclic 1,2,3-triazole derivatives *via* a domino reaction (Pokhodylo & Shyyka, 2017 ▸p; Pokhodylo *et al.*, 2014 ▸), but for reagents with labile functional groups (Pokhodylo *et al.*, 2018 ▸, 2020 ▸) or to avoid concurrent Regitz diazo­transfer reaction (Pokhodylo & Obushak, 2019 ▸), mild bases such as K_2_CO_3_ (Pokhodylo *et al.*, 2017 ▸) or organic bases (Et_3_N, DBU, pyrrolidine) are more suitable (Blastik *et al.*, 2018 ▸; Ramachary *et al.*, 2008 ▸; Danence *et al.*, 2011 ▸). Furthermore, it has been shown that mild bases Et_3_N could be used for regioselective introduction of strongly electron-withdrawing groups such as tri­fluoro­iodo­methyl (CF_3_) in the 1,2,3-triazole ring in the reaction with asymmetric 1,3-diketones (Rozin *et al.*, 2012 ▸).

Taking into account the above facts, in this work, the title compound, (I), was obtained and its crystal structure determined. 
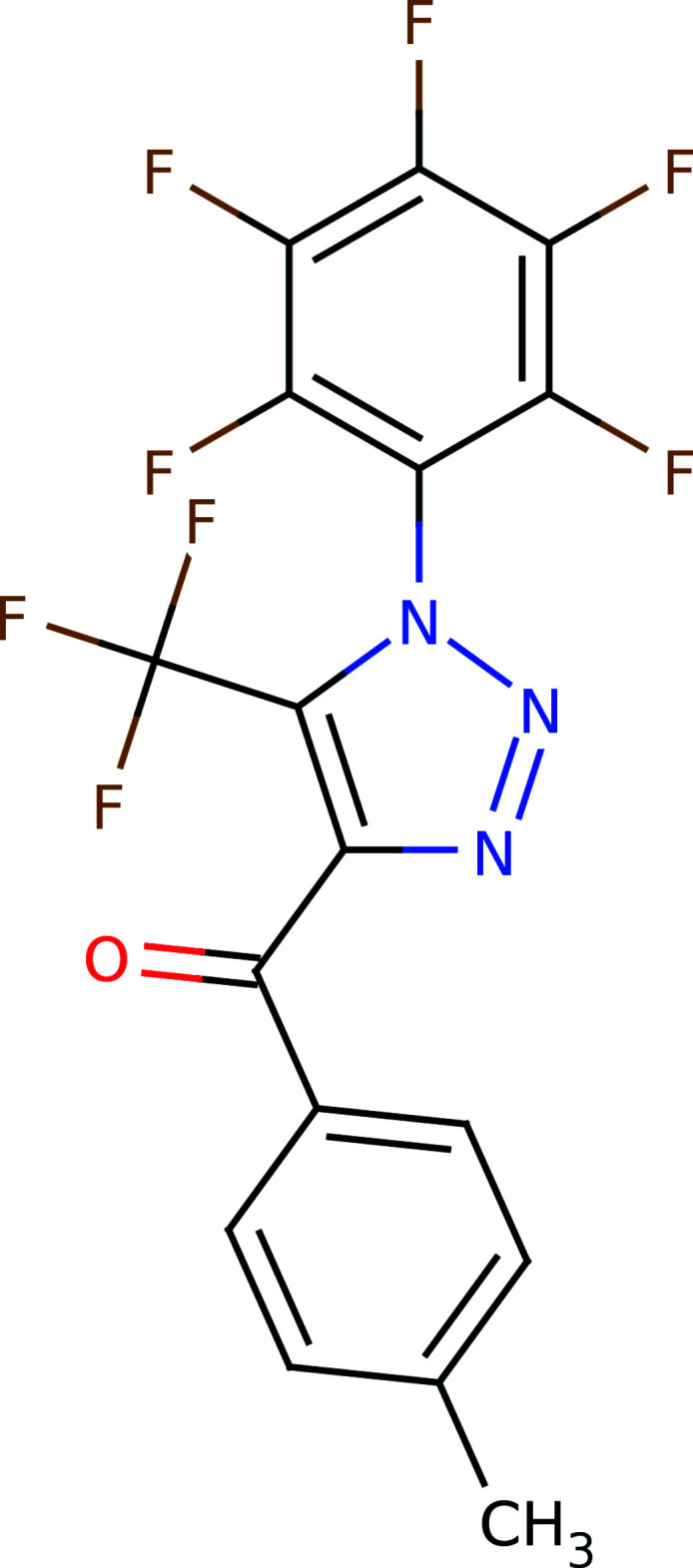


## Structural commentary

**Figure 1:**
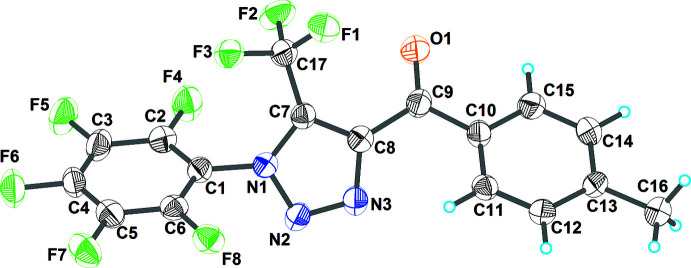
The mol­ecular structure of (I) with displacement ellipsoids drawn at the 50% probability level.

The title compound crystallizes in the non-centrosymmetric space group *P*2_1_2_1_2_1_, with one mol­ecule in the asymmetric unit. As shown in Fig. 1 ▸, it is constructed from three aromatic rings (C10–C15 4-methyl­phenyl, C1–C6 penta­fluoro­phenyl and C7/C8/N1/N2/N3 triazole rings). The penta­fluoro­phenyl ring and the heterocyclic ring are twisted relative to each other by 62.3 (2)° because of the significant steric hindrance of the trifluoromethyl group attached to C7. This dihedral angle is significantly smaller than the angle of 87.1° between the 4-nitro­phenyl and triazole rings in the structure of 1-[5-methyl-1-(4-nitro­phen­yl)-1*H*-1,2,3-triazol-4-yl]ethanone (VI) (Vinutha *et al.*, 2013 ▸) but considerably larger than the analogous angle between aromatic rings in the structures of 3-(4-fluoro­phen­yl)-1-[1-(4-fluoro­phen­yl)-5-methyl-1*H*-1,2,3-tri­az­ol-4-yl]prop-2-en-1-one (39.6°; El-Hiti *et al.*, 2018 ▸), (4-meth­yl­phen­yl)(5-methyl-1-phenyl-1*H*-1,2,3-triazol-4-yl)methanone (44.5°; Li *et al.*, 2014 ▸), 1-[1-(4-chloro­phen­yl)-5-methyl-1*H*-1,2,3-triazol-4-yl]ethanone (45.6°) and 1-[1-(4-bromo­phen­yl)-5-methyl-1*H*-1,2,3-triazol-4-yl]ethanone (47.1°) (Zeghada *et al.*, 2011 ▸). The carbonyl group of the title compound is not in the plane of the adjacent aromatic rings: the C7—C8—C9—O1 and C15—C10—C9—O1 torsion angles are −25.4 (9) and −16.8 (9)°, respectively]. The 4-methyl­phenyl and triazole rings are twisted relative to each other by 43.9 (2)° and the 4-methyl­phenyl and penta­fluoro­phenyl rings by 19.1 (3)°.

## Supra­molecular features

**Figure 2:**
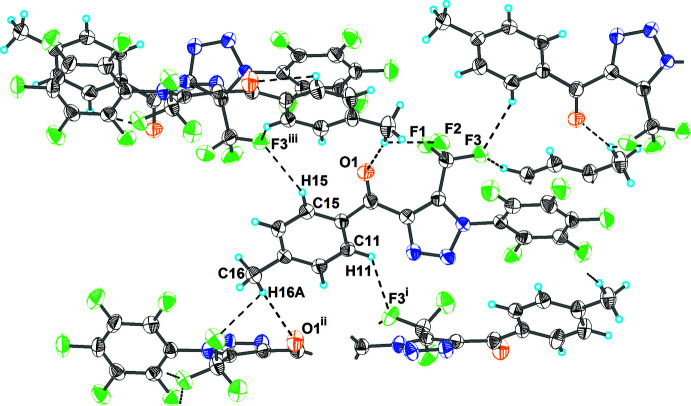
The hydrogen bonding of mol­ecules in (I). Hydrogen bonds are shown as dashed lines. The symmetry codes are as in Table 1 ▸.

**Figure 3:**
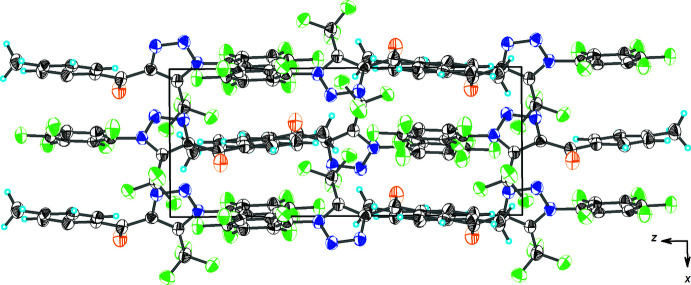
A view along the *b*-axis direction of the crystal packing of (I).

As shown in Fig. 2 ▸ and listed in Table 1 ▸, the crystal structure of (I) features several weak inter­molecular inter­actions. The hydrogen atoms of the 4-methyl­phenyl ring are involved in C—H⋯F hydrogen bonding with the tri­fluoro­methyl substituents of adjacent mol­ecules, while a hydrogen atom of the methyl group forms a C—H⋯O hydrogen bond with the carbonyl O atom of another adjacent mol­ecule. The 4-methyl­phenyl and penta­fluoro­phenyl rings of adjacent mol­ecules are also involved into face-to-face π–π stacking inter­action with a centroid–centroid separation of 3.783 (6) Å, while at the same time the triazole rings are involved into edge-to-face aromatic inter­actions at 3.218 (6) Å. The mol­ecules are linked by the above-mentioned inter­molecular inter­actions into a three-dimensional network (Fig. 3 ▸).

## Hirshfeld surface analysis

**Figure 4:**
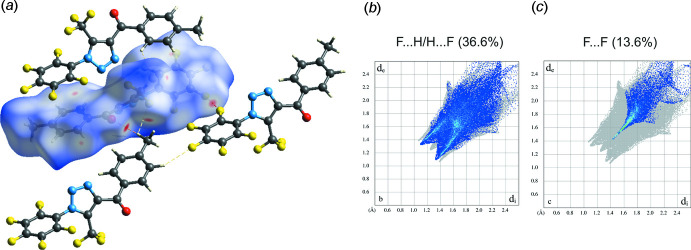
(*a*) Hirshfeld surface for (I) mapped with *d*_norm_ over the range −0.12 to 1.53 a.u. showing C—H⋯O and C—H⋯F hydrogen-bonded contacts as well as F⋯F contacts. Fingerprint plots resolved into (*b*) F⋯H/H⋯F and (*c*) F⋯F contacts. Neighbouring mol­ecules associated with close contacts are also shown.

Hirshfeld surface analysis was used to analyse the various inter­molecular inter­actions in (I), through mapping the normalized contact distance (*d*_norm_) using *CrystalExplorer* (Turner *et al.*, 2017 ▸; Spackman & Jayatilaka, 2009 ▸). The most prominent inter­actions (bifurcated inter­actions of atom H16*A* of the methyl group with the carbonyl group O atom and the fluorine atom of the tri­fluoro­methyl substituent of neighbouring mol­ecules, as well as the F⋯F inter­action between neighbouring penta­fluoro­phenyl rings) can be seen in the Hirshfeld surface plot as red areas (Fig. 4 ▸). Fingerprint plots were produced to show the inter­molecular surface bond distances with the regions highlighted for F⋯H/H⋯F and F⋯F contacts inter­actions (Fig. 4 ▸). The contribution to the surface area for such contacts are 36.6% and 13.6%, respectively. The contribution to the surface area for O⋯H/H⋯O and H⋯H contacts are 4.6% and 5.7%, respectively.

## Database survey

The most closely related compounds, containing a similar 1-aryl-1*H-*1,2,3-triazole-4-carbonyl skeleton to the title compound but with different substituents on the carbonyl group are: 2,2′-(quinoxaline-2,3-diyldisulfanedi­yl)bis­{1-[5-methyl-1-(4-methyl­phen­yl)-1*H*-1,2,3-triazol-4-yl]ethan-1-one} (II) [Cambridge Structural Database (Version 2021.1; Groom *et al.*, 2016 ▸) refcode ETUVEX; Mohamed *et al.*, 2021 ▸], 4-(4-acetyl-5-methyl-1*H*-1,2,3-triazol-1-yl)benzo­nitrile (III) (SILBOH; Zukerman-Schpector *et al.*, 2018 ▸), 1-[5-methyl-1-(4-methyl­phen­yl)-1*H*-1,2,3-triazol-4-yl]ethan-1-one (IV) (LEMSUU; El-Hiti *et al.*, 2017 ▸), 3-(4-fluoro­phen­yl)-1-[1-(4-fluoro­phen­yl)-5-methyl-1*H*-1,2,3-triaz­ol-4-yl]prop-2-en-1-one (V) (MESTAI; El-Hiti *et al.*, 2018 ▸), 1-[5-methyl-1-(4-nitro­phen­yl)-1*H*-1,2,3-triazol-4-yl]ethanone (VI) (QIRQOZ; Vinutha *et al.*, 2013 ▸), 2-bromo-1-[1-(4-bromo­phen­yl)-5-methyl-1*H*-1,2,3-triazol-4-yl]ethanone (VII) (XODSAM; Bunev *et al.*, 2014 ▸), (4-methyl­phen­yl)(5-methyl-1-phenyl-1*H*-1,2,3-triazol-4-yl)methanone (VIII) (COCYAW; Li *et al.*, 2014 ▸), (2*E*)-3-(4-fluoro­phen­yl)-1-[5-methyl-1-(4-methyl­phen­yl)-1*H*-1,2,3-triazol-4-yl]prop-2-en-1-one (IX) (IDITUM; Abdel-Wahab *et al.*, 2013 ▸), 1-[1-(4-chloro­phen­yl)-5-methyl-1*H*-1,2,3-triazol-4-yl]ethanone (X) (ISOBUO; Zeghada *et al.*, 2011 ▸) and 1-[1-(4-bromo­phen­yl)-5-methyl-1*H*-1,2,3-triazol-4-yl]ethanone (XI) (ISOCAV; Zeghada *et al.*, 2011 ▸).

Compounds (V), (IX), (X) and (XI) crystallize in the triclinic crystal system in space group *P* 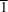 . Compounds (II), (III) and (IV), (VIII) crystallize in the monoclinic crystal system with space groups *P*2_1_/*n* and *P*2_1_/*c*, respectively, while compound (VII) is found in the monoclinic crystal system, space group *Pn*. Compound (VI) crystallizes in the ortho­rhom­bic crystal system in non-centrosymmetric space group *Pca*2_1_. Structures (V), (VI) and (VII) contain two crystallographically independent mol­ecules. The aryl and triazole rings in (VI) are twisted relative to each other by 87.1 and 38.2° in the two crystallographically independent mol­ecules. In compounds (III), (IV) and (IX), the analogous angles between the aromatic rings are 54.7, 50.1 and 51.8°, respectively.

## Synthesis and crystallization

**Figure 5:**
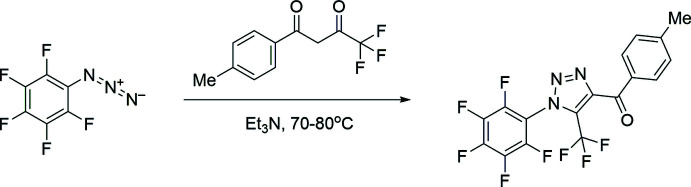
Synthesis scheme for (I)

A number of experimental conditions described previously were investigated for the synthesis of the title compound (Shafran *et al.*, 2019 ▸; Pokhodylo *et al.*, 2017 ▸; Blastik *et al.*, 2018 ▸; Rozin *et al.*, 2012 ▸). However, it was possible to obtain the target product only in the case of the protocol proposed by Rozin *et al.* (2012 ▸). The synthesis scheme is shown in Fig. 5 ▸.

**(4-Methyl­phen­yl)[1-(penta­fluoro­phen­yl)-5-(tri­fluoro­meth­yl)-1** ***H*** **-1,2,3-triazol-4-yl]methanone**: A mixture of the corres­ponding 4,4,4-tri­fluoro-1-(*p*-tol­yl)butane-1,3-dione 230 mg (1.00 mmol), 1-azido-2,3,4,5,6-penta­fluoro­benzene 209 mg (1.00 mmol), and tri­ethyl­amine (0.43 ml, 3.00 mmol) was heated at 343–353 K for 3 h. Volatiles were evaporated *in vacuo* and the residue was purified by column chromatography on silica gel using di­chloro­methane as an eluent. Colourless crystals were grown by slow evaporation of a di­chloro­methane solution, yield 21%; m.p. 391–394 K; ^1^H NMR (500 MHz, DMSO-*d*_6_) δ 8.02 (*d*, *J* = 7.9 Hz, 2H, H^Ar^-2,6), 7.44 (*d*, *J* = 7.8 Hz, 2H, H^Ar^-3,5), 2.43 (*s*, 3H); ^13^C NMR (126 MHz, DMSO-*d*_6_) δ 183.74 (CO), 145.73 (C^Tol^-4), 145.57 (C^Triazole^-4), 143.61 (*m*), 141.68 (*m*), 138.84 (*m*), 136.79 (*m*), 132.61 (C^Tol^-1), 130.67 (*q*, *J* = 41.7 Hz, C^Triazole^-5), 130.63 (2 × C^Tol^-2,6), 129.49 (2 × C^Tol^-3,5), 118.36 (*q*, ^1^ *J*_C–F_ = 270.9 Hz, CF_3_), 109.59 (*m*), 21.37 (CH_3_); ^19^F NMR (376 MHz, DMSO-*d*_6_) δ −58.46 (CF_3_), −146.39 (*d*, *J* = 21.5 Hz, 2 × F-2,6), −146.53 (*t*, *J* = 23.4 Hz, F-4), −159.61 (*t*, *J* = 23.3 Hz, 2 × F-3,5); MS, *m*/*z* = 422 (*M* ^+^ + 1). Calculated for C_17_H_7_F_8_N_3_O, (%): C, 48.47; H, 1.68; N, 9.98. Found (%): C, 48.55; H, 1.67; N, 9.91.

## Refinement

Crystal data, data collection and structure refinement details are summarized in Table 2 ▸. All H atoms were positioned geometrically with C—H = 0.95–0.98 Å and refined as riding atoms. The constraint *U*_iso_(H) = 1.2*U*_eq_(carrier) or 1.5*U*_eq_(C-methyl carrier) was applied in all cases.

## Supplementary Material

Crystal structure: contains datablock(s) I, global. DOI: 10.1107/S2056989021010070/hb7985sup1.cif

Structure factors: contains datablock(s) I. DOI: 10.1107/S2056989021010070/hb7985Isup2.hkl

Supporting information file. DOI: 10.1107/S2056989021010070/hb7985Isup4.cml

NMR spectra. DOI: 10.1107/S2056989021010070/hb7985sup3.pdf

CCDC reference: 2112438

Additional supporting information:  crystallographic information; 3D view; checkCIF report

## Figures and Tables

**Table 1:** Hydrogen-bond geometry (Å, °)

*D*—H⋯*A*	*D*—H	H⋯*A*	*D*⋯*A*	*D*—H⋯*A*
C11—H11⋯F3^i^	0.95	2.49	3.155 (5)	127
C15—H15⋯F3^ii^	0.95	2.61	3.463 (6)	149
C16—H16*A*⋯F2^iii^	0.98	2.57	3.054 (6)	111
C16—H16*A*⋯O1^iii^	0.98	2.54	3.505 (6)	167

Symmetry codes: (i) x+{\script{1\over 2}}, -y+{\script{1\over 2}}, -z+1; (ii) -x+{\script{1\over 2}}, -y+1, z+{\script{1\over 2}}; (iii) -x+1, y-{\script{1\over 2}}, -z+{\script{3\over 2}}.

**Table 2:** Experimental details

Crystal data	
Chemical formula	C_17_H_7_F_8_N_3_O
*M*_r_	421.26
Crystal system, space group	Orthorhombic, *P*2_1_2_1_2_1_
Temperature (K)	150
*a*, *b*, *c* (Å)	6.7605 (6), 15.065 (1), 16.0849 (9)
*V* (Å^3^)	1638.2 (2)
*Z*	4
Radiation type	Cu *K*α
μ (mm^−1^)	1.55
Crystal size (mm)	0.43 × 0.12 × 0.08

Data collection	
Diffractometer	New Gemini, Dual, Cu at home/near, Atlas
Absorption correction	Analytical (*CrysAlis PRO*; Rigaku OD, 2021 ▸)
*T*_min_, *T*_max_	0.320, 0.678
No. of measured, independent and observed [*I* > 2σ(*I*)] reflections	15249, 3187, 2311
*R*_int_	0.088
(sin θ/λ)_max_ (Å^−1^)	0.618

Refinement	
*R*[*F* ^2^ > 2σ(*F* ^2^)], *wR*(*F* ^2^), *S*	0.047, 0.109, 1.03
No. of reflections	3187
No. of parameters	264
H-atom treatment	H-atom parameters constrained
Δρ_max_, Δρ_min_ (e Å^−3^)	0.18, −0.18
Absolute structure	Refined as an inversion twin.
Absolute structure parameter	0.2 (3)

Computer programs: *CrysAlis PRO* (Rigaku OD, 2021 ▸), *SHELXT* (Sheldrick, 2015*a* ▸), *SHELXL2018/3* (Sheldrick, 2015*b* ▸) and *OLEX2* (Dolomanov *et al.*, 2009 ▸).

## supplementary crystallographic information

### Crystal data

**Table:**

C_17_H_7_F_8_N_3_O	*D*_x_ = 1.708 Mg m^−^^3^
*M**_r_* = 421.26	Cu *K*α radiation, λ = 1.54184 Å
Orthorhombic, *P*2_1_2_1_2_1_	Cell parameters from 2646 reflections
*a* = 6.7605 (6) Å	θ = 4.0–72.2°
*b* = 15.065 (1) Å	µ = 1.55 mm^−^^1^
*c* = 16.0849 (9) Å	*T* = 150 K
*V* = 1638.2 (2) Å^3^	Irregular, colourless
*Z* = 4	0.43 × 0.12 × 0.08 mm
*F*(000) = 840	

### Data collection

**Table:**

New Gemini, Dual, Cu at home/near, Atlas diffractometer	2311 reflections with *I* > 2σ(*I*)
Detector resolution: 10.6426 pixels mm^-1^	*R*_int_ = 0.088
ω scans	θ_max_ = 72.3°, θ_min_ = 4.0°
Absorption correction: analytical (CrysalisPro; Rigaku OD, 2021)	*h* = −7→8
*T*_min_ = 0.320, *T*_max_ = 0.678	*k* = −18→18
15249 measured reflections	*l* = −19→19
3187 independent reflections	

### Refinement

**Table:**

Refinement on *F*^2^	Hydrogen site location: inferred from neighbouring sites
Least-squares matrix: full	H-atom parameters constrained
*R*[*F*^2^ > 2σ(*F*^2^)] = 0.047	*w* = 1/[σ^2^(*F*_o_^2^) + (0.031*P*)^2^ + 0.1487*P*] where *P* = (*F*_o_^2^ + 2*F*_c_^2^)/3
*wR*(*F*^2^) = 0.109	(Δ/σ)_max_ < 0.001
*S* = 1.03	Δρ_max_ = 0.18 e Å^−^^3^
3187 reflections	Δρ_min_ = −0.18 e Å^−^^3^
264 parameters	Absolute structure: Refined as an inversion twin.
0 restraints	Absolute structure parameter: 0.2 (3)

### Special details

**Table:**

Geometry. All esds (except the esd in the dihedral angle between two l.s. planes) are estimated using the full covariance matrix. The cell esds are taken into account individually in the estimation of esds in distances, angles and torsion angles; correlations between esds in cell parameters are only used when they are defined by crystal symmetry. An approximate (isotropic) treatment of cell esds is used for estimating esds involving l.s. planes.
Refinement. Refined as a 2-component inversion twin.

### Fractional atomic coordinates and isotropic or equivalent isotropic displacement parameters (Å^2^)

**Table:**

	*x*	*y*	*z*	*U*_iso_*/*U*_eq_	
F1	0.0932 (5)	0.4231 (2)	0.51007 (19)	0.0563 (9)	
F2	0.2755 (6)	0.52826 (18)	0.4634 (2)	0.0575 (10)	
F3	0.1757 (5)	0.42585 (19)	0.38129 (17)	0.0456 (8)	
F4	0.5987 (6)	0.50612 (18)	0.33789 (17)	0.0565 (9)	
F5	0.6160 (6)	0.5157 (2)	0.16979 (18)	0.0609 (10)	
F6	0.5614 (6)	0.3674 (3)	0.07762 (16)	0.0612 (10)	
F7	0.4985 (6)	0.2089 (2)	0.15264 (18)	0.0566 (9)	
F8	0.4847 (5)	0.19857 (17)	0.32018 (16)	0.0456 (8)	
O1	0.3633 (7)	0.4664 (2)	0.6435 (2)	0.0496 (10)	
N1	0.5368 (7)	0.3474 (2)	0.4207 (2)	0.0351 (10)	
N2	0.6809 (7)	0.2985 (3)	0.4582 (2)	0.0418 (11)	
N3	0.6565 (8)	0.3072 (3)	0.5387 (2)	0.0410 (10)	
C1	0.5367 (8)	0.3525 (3)	0.3316 (3)	0.0339 (11)	
C2	0.5726 (8)	0.4330 (3)	0.2930 (3)	0.0396 (12)	
C3	0.5793 (9)	0.4382 (4)	0.2066 (3)	0.0451 (13)	
C4	0.5531 (9)	0.3623 (4)	0.1611 (3)	0.0430 (13)	
C5	0.5215 (9)	0.2822 (3)	0.1983 (3)	0.0405 (12)	
C6	0.5130 (8)	0.2771 (3)	0.2847 (3)	0.0357 (11)	
C7	0.4210 (8)	0.3878 (3)	0.4781 (3)	0.0319 (11)	
C8	0.4981 (8)	0.3615 (3)	0.5531 (3)	0.0337 (11)	
C9	0.4375 (9)	0.3934 (3)	0.6379 (3)	0.0385 (13)	
C10	0.4732 (8)	0.3363 (3)	0.7117 (3)	0.0345 (11)	
C11	0.5049 (8)	0.2451 (3)	0.7063 (3)	0.0340 (10)	
H11	0.509418	0.217046	0.653465	0.041*	
C12	0.5297 (8)	0.1955 (3)	0.7776 (3)	0.0360 (11)	
H12	0.549256	0.133197	0.773331	0.043*	
C13	0.5267 (8)	0.2352 (3)	0.8560 (3)	0.0374 (12)	
C14	0.4964 (10)	0.3264 (3)	0.8609 (3)	0.0454 (14)	
H14	0.494442	0.354584	0.913774	0.054*	
C15	0.4693 (9)	0.3765 (3)	0.7897 (3)	0.0438 (14)	
H15	0.447771	0.438645	0.794080	0.053*	
C16	0.5547 (10)	0.1808 (3)	0.9339 (3)	0.0493 (16)	
H16A	0.562191	0.117776	0.919256	0.074*	
H16B	0.442677	0.190607	0.971434	0.074*	
H16C	0.677506	0.198827	0.961531	0.074*	
C17	0.2417 (9)	0.4415 (3)	0.4588 (3)	0.0405 (12)	

### Atomic displacement parameters (Å^2^)

**Table:**

	*U*^11^	*U*^22^	*U*^33^	*U*^12^	*U*^13^	*U*^23^
F1	0.045 (2)	0.0728 (19)	0.0511 (16)	0.0166 (18)	0.0065 (16)	0.0039 (15)
F2	0.077 (3)	0.0330 (13)	0.0620 (18)	0.0102 (16)	−0.0228 (18)	−0.0058 (14)
F3	0.049 (2)	0.0459 (15)	0.0416 (14)	0.0086 (15)	−0.0134 (14)	−0.0077 (12)
F4	0.084 (3)	0.0395 (15)	0.0464 (15)	−0.0048 (17)	0.0019 (16)	0.0027 (13)
F5	0.073 (3)	0.0606 (18)	0.0492 (16)	−0.001 (2)	0.0050 (17)	0.0228 (15)
F6	0.061 (3)	0.094 (2)	0.0285 (13)	0.005 (2)	−0.0016 (15)	0.0040 (14)
F7	0.058 (2)	0.0650 (18)	0.0470 (15)	−0.001 (2)	−0.0009 (17)	−0.0199 (14)
F8	0.050 (2)	0.0372 (13)	0.0497 (15)	−0.0019 (16)	0.0064 (15)	−0.0027 (11)
O1	0.072 (3)	0.0354 (17)	0.0412 (18)	0.0107 (19)	0.0010 (19)	0.0006 (14)
N1	0.040 (3)	0.0344 (18)	0.0312 (17)	0.005 (2)	−0.0025 (19)	0.0008 (15)
N2	0.042 (3)	0.049 (2)	0.0345 (19)	0.008 (2)	0.0005 (19)	0.0094 (19)
N3	0.042 (3)	0.050 (2)	0.0316 (19)	0.004 (2)	0.0010 (19)	0.0040 (17)
C1	0.031 (3)	0.041 (2)	0.030 (2)	0.004 (2)	−0.002 (2)	0.0003 (18)
C2	0.042 (3)	0.038 (2)	0.039 (2)	0.004 (3)	−0.001 (2)	0.000 (2)
C3	0.046 (3)	0.053 (3)	0.037 (2)	0.001 (3)	0.003 (2)	0.016 (2)
C4	0.037 (4)	0.067 (3)	0.026 (2)	0.008 (3)	0.002 (2)	0.002 (2)
C5	0.030 (3)	0.053 (3)	0.038 (2)	0.002 (3)	−0.003 (2)	−0.008 (2)
C6	0.026 (3)	0.039 (2)	0.041 (2)	0.002 (2)	0.002 (2)	0.0021 (19)
C7	0.037 (3)	0.0276 (19)	0.031 (2)	−0.002 (2)	0.000 (2)	0.0012 (17)
C8	0.032 (3)	0.034 (2)	0.035 (2)	−0.002 (2)	0.000 (2)	0.0018 (17)
C9	0.046 (4)	0.037 (2)	0.032 (2)	0.002 (2)	−0.003 (2)	0.0021 (19)
C10	0.036 (3)	0.039 (2)	0.0285 (19)	0.001 (2)	0.000 (2)	0.0009 (17)
C11	0.030 (3)	0.039 (2)	0.033 (2)	0.002 (2)	0.000 (2)	−0.0025 (17)
C12	0.034 (3)	0.038 (2)	0.036 (2)	0.003 (2)	0.001 (2)	0.0006 (18)
C13	0.035 (3)	0.045 (2)	0.032 (2)	0.009 (2)	−0.004 (2)	0.0030 (18)
C14	0.058 (4)	0.047 (3)	0.031 (2)	0.011 (3)	−0.001 (3)	−0.0058 (19)
C15	0.049 (4)	0.045 (3)	0.037 (2)	0.012 (3)	−0.007 (3)	−0.005 (2)
C16	0.061 (5)	0.052 (3)	0.036 (2)	0.014 (3)	−0.001 (3)	0.005 (2)
C17	0.050 (4)	0.038 (2)	0.034 (2)	0.007 (3)	−0.006 (2)	−0.004 (2)

### Geometric parameters (Å, º)

**Table:**

F1—C17	1.328 (7)	C5—C6	1.393 (6)
F2—C17	1.329 (6)	C7—C8	1.373 (6)
F3—C17	1.345 (6)	C7—C17	1.490 (8)
F4—C2	1.328 (6)	C8—C9	1.502 (6)
F5—C3	1.332 (6)	C9—C10	1.485 (6)
F6—C4	1.346 (5)	C10—C11	1.393 (6)
F7—C5	1.336 (6)	C10—C15	1.394 (6)
F8—C6	1.328 (5)	C11—H11	0.9500
O1—C9	1.213 (6)	C11—C12	1.380 (6)
N1—N2	1.362 (6)	C12—H12	0.9500
N1—C1	1.435 (5)	C12—C13	1.395 (6)
N1—C7	1.355 (6)	C13—C14	1.392 (7)
N2—N3	1.313 (6)	C13—C16	1.508 (6)
N3—C8	1.368 (7)	C14—H14	0.9500
C1—C2	1.384 (7)	C14—C15	1.384 (7)
C1—C6	1.373 (7)	C15—H15	0.9500
C2—C3	1.393 (7)	C16—H16A	0.9800
C3—C4	1.369 (8)	C16—H16B	0.9800
C4—C5	1.363 (8)	C16—H16C	0.9800
			
N2—N1—C1	118.1 (4)	C10—C9—C8	119.7 (4)
C7—N1—N2	110.8 (4)	C11—C10—C9	123.1 (4)
C7—N1—C1	131.0 (4)	C11—C10—C15	119.1 (4)
N3—N2—N1	107.1 (4)	C15—C10—C9	117.7 (4)
N2—N3—C8	109.0 (4)	C10—C11—H11	119.9
C2—C1—N1	119.6 (4)	C12—C11—C10	120.1 (4)
C6—C1—N1	120.4 (4)	C12—C11—H11	119.9
C6—C1—C2	119.9 (4)	C11—C12—H12	119.4
F4—C2—C1	120.4 (4)	C11—C12—C13	121.1 (4)
F4—C2—C3	119.4 (5)	C13—C12—H12	119.4
C1—C2—C3	120.1 (5)	C12—C13—C16	121.0 (4)
F5—C3—C2	119.9 (5)	C14—C13—C12	118.5 (4)
F5—C3—C4	121.2 (4)	C14—C13—C16	120.5 (4)
C4—C3—C2	118.9 (5)	C13—C14—H14	119.6
F6—C4—C3	118.7 (5)	C15—C14—C13	120.7 (4)
F6—C4—C5	119.7 (5)	C15—C14—H14	119.6
C5—C4—C3	121.6 (4)	C10—C15—H15	119.8
F7—C5—C4	120.6 (4)	C14—C15—C10	120.4 (4)
F7—C5—C6	119.8 (5)	C14—C15—H15	119.8
C4—C5—C6	119.6 (5)	C13—C16—H16A	109.5
F8—C6—C1	121.1 (4)	C13—C16—H16B	109.5
F8—C6—C5	119.0 (4)	C13—C16—H16C	109.5
C1—C6—C5	119.9 (4)	H16A—C16—H16B	109.5
N1—C7—C8	104.5 (4)	H16A—C16—H16C	109.5
N1—C7—C17	124.9 (4)	H16B—C16—H16C	109.5
C8—C7—C17	130.4 (4)	F1—C17—F2	107.5 (4)
N3—C8—C7	108.7 (4)	F1—C17—F3	106.8 (5)
N3—C8—C9	123.9 (4)	F1—C17—C7	111.9 (4)
C7—C8—C9	127.0 (5)	F2—C17—F3	106.3 (4)
O1—C9—C8	118.0 (4)	F2—C17—C7	112.5 (5)
O1—C9—C10	122.2 (4)	F3—C17—C7	111.6 (4)
			
F4—C2—C3—F5	1.5 (9)	C2—C1—C6—F8	177.8 (5)
F4—C2—C3—C4	179.7 (5)	C2—C1—C6—C5	−1.2 (9)
F5—C3—C4—F6	−1.2 (9)	C2—C3—C4—F6	−179.4 (5)
F5—C3—C4—C5	178.0 (6)	C2—C3—C4—C5	−0.3 (10)
F6—C4—C5—F7	−0.1 (9)	C3—C4—C5—F7	−179.3 (6)
F6—C4—C5—C6	180.0 (5)	C3—C4—C5—C6	0.9 (10)
F7—C5—C6—F8	0.9 (9)	C4—C5—C6—F8	−179.2 (5)
F7—C5—C6—C1	−180.0 (5)	C4—C5—C6—C1	−0.1 (9)
O1—C9—C10—C11	161.5 (6)	C6—C1—C2—F4	−179.0 (5)
O1—C9—C10—C15	−16.8 (9)	C6—C1—C2—C3	1.8 (9)
N1—N2—N3—C8	−0.2 (6)	C7—N1—N2—N3	0.4 (6)
N1—C1—C2—F4	−2.9 (9)	C7—N1—C1—C2	61.7 (8)
N1—C1—C2—C3	177.9 (5)	C7—N1—C1—C6	−122.2 (6)
N1—C1—C6—F8	1.8 (9)	C7—C8—C9—O1	−25.4 (9)
N1—C1—C6—C5	−177.3 (5)	C7—C8—C9—C10	155.8 (5)
N1—C7—C8—N3	0.3 (5)	C8—C7—C17—F1	−37.5 (7)
N1—C7—C8—C9	174.0 (5)	C8—C7—C17—F2	83.6 (7)
N1—C7—C17—F1	136.6 (5)	C8—C7—C17—F3	−157.0 (5)
N1—C7—C17—F2	−102.3 (5)	C8—C9—C10—C11	−19.9 (9)
N1—C7—C17—F3	17.1 (7)	C8—C9—C10—C15	161.9 (5)
N2—N1—C1—C2	−114.2 (6)	C9—C10—C11—C12	−177.5 (5)
N2—N1—C1—C6	61.9 (7)	C9—C10—C15—C14	178.3 (6)
N2—N1—C7—C8	−0.4 (5)	C10—C11—C12—C13	−0.9 (9)
N2—N1—C7—C17	−175.8 (4)	C11—C10—C15—C14	0.0 (9)
N2—N3—C8—C7	−0.1 (6)	C11—C12—C13—C14	0.5 (9)
N2—N3—C8—C9	−174.1 (5)	C11—C12—C13—C16	−179.7 (6)
N3—C8—C9—O1	147.4 (5)	C12—C13—C14—C15	0.2 (10)
N3—C8—C9—C10	−31.3 (8)	C13—C14—C15—C10	−0.5 (10)
C1—N1—N2—N3	177.1 (4)	C15—C10—C11—C12	0.7 (9)
C1—N1—C7—C8	−176.5 (5)	C16—C13—C14—C15	−179.5 (6)
C1—N1—C7—C17	8.1 (8)	C17—C7—C8—N3	175.3 (5)
C1—C2—C3—F5	−179.3 (5)	C17—C7—C8—C9	−11.0 (9)
C1—C2—C3—C4	−1.1 (10)		

### Hydrogen-bond geometry (Å, º)

**Table:**

*D*—H···*A*	*D*—H	H···*A*	*D*···*A*	*D*—H···*A*
C11—H11···F3^i^	0.95	2.49	3.155 (5)	127
C15—H15···F3^ii^	0.95	2.61	3.463 (6)	149
C16—H16*A*···F2^iii^	0.98	2.57	3.054 (6)	111
C16—H16*A*···O1^iii^	0.98	2.54	3.505 (6)	167

Symmetry codes: (i) *x*+1/2, −*y*+1/2, −*z*+1; (ii) −*x*+1/2, −*y*+1, *z*+1/2; (iii) −*x*+1, *y*−1/2, −*z*+3/2.
