# Supplementary material for: Synthesis, crystal structure and Hirshfeld surface analysis of (4-methyl­phen­yl)[1-(penta­fluoro­phen­yl)-5-(tri­fluoro­meth­yl)-1H-1,2,3-triazol-4-yl]methanone
Source: Acta Crystallogr E Crystallogr Commun. 2021 Oct 5;77(Pt 11):1067–71. doi: 10.1107/S2056989021010070 (PMC8587975; doi:10.1107/S2056989021010070)

^{1}H NMR (1-(Perfluorophenyl)-5-(trifluoromethyl)-1H-1,2,3-triazol-4-yl)(p-tolyl)methanone

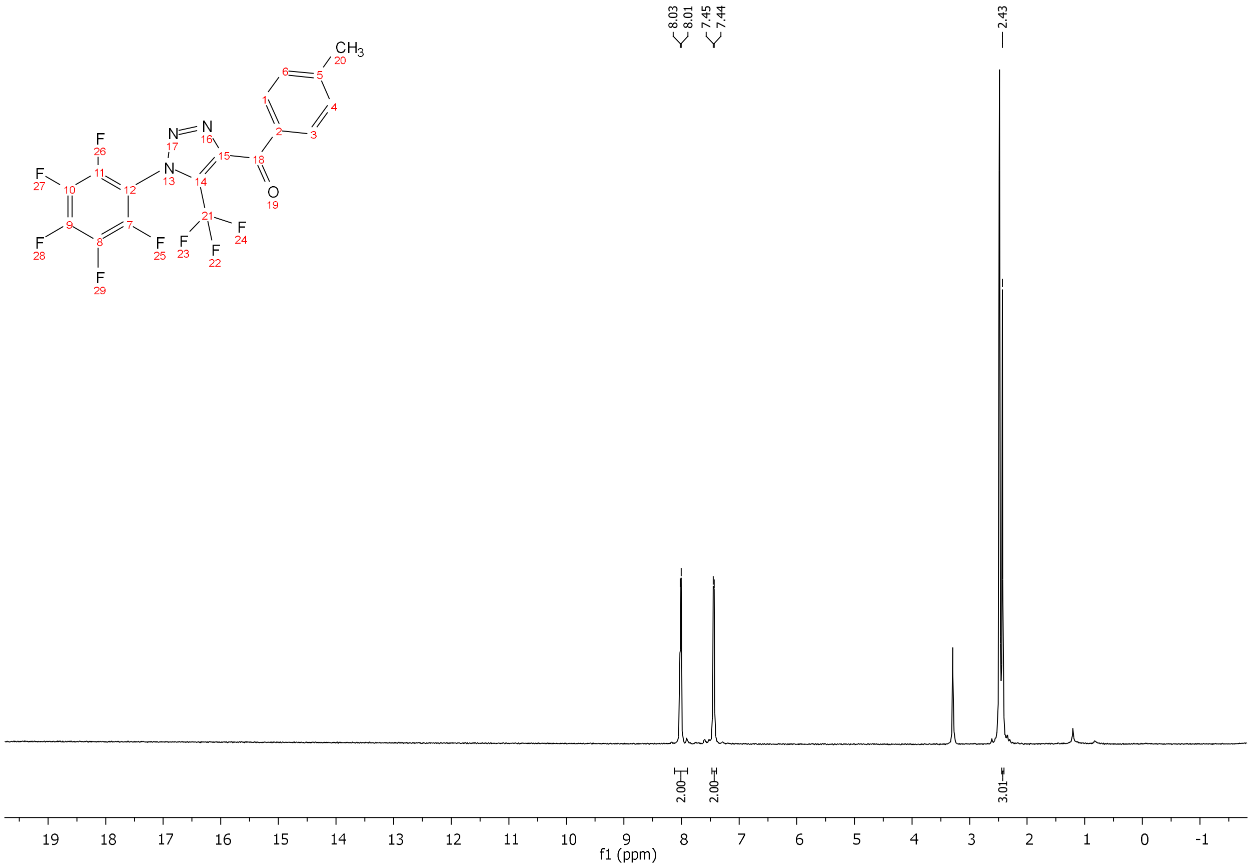

^{13}C NMR (1-(Perfluorophenyl)-5-(trifluoromethyl)-1H-1,2,3-triazol-4-yl)(p-tolyl)methanone

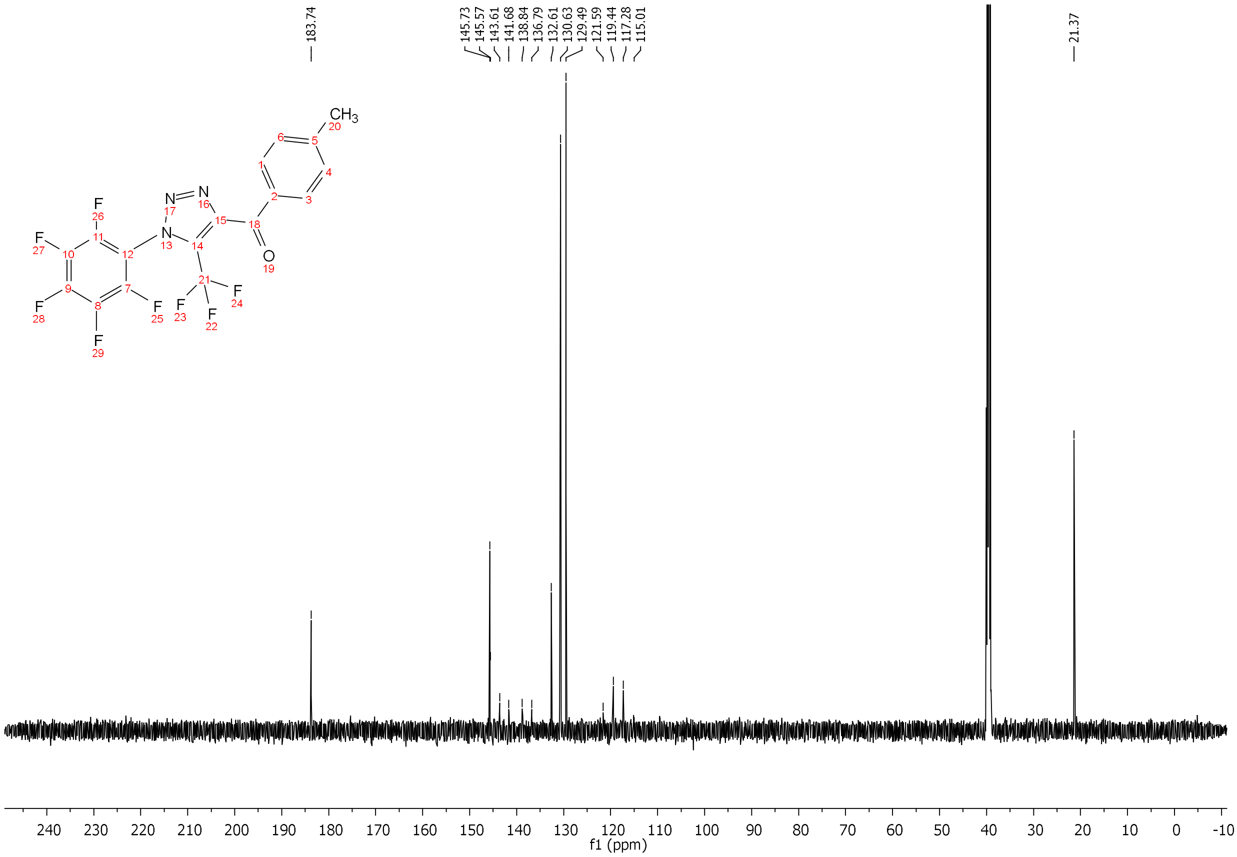

^{19}F NMR (1-(Perfluorophenyl)-5-(trifluoromethyl)-1H-1,2,3-triazol-4-yl)(p-tolyl)methanone

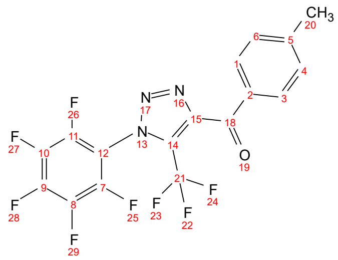

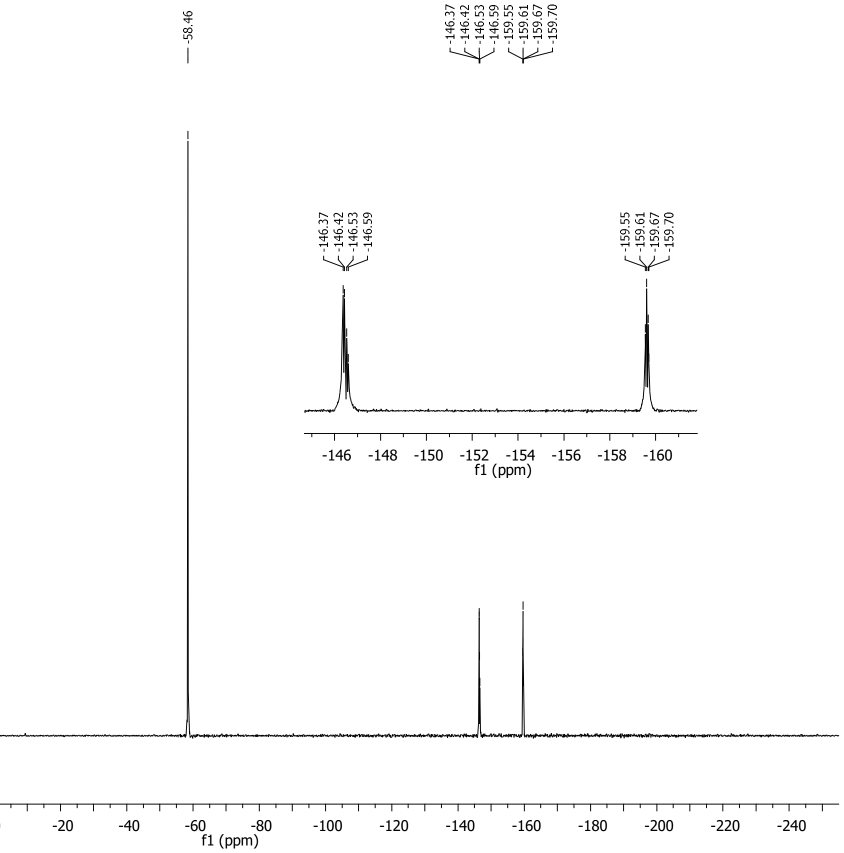

Supplement: Supplementary file 4 [file e-77-01067-sup3.pdf]
